# Gut Microbiota of *Apis mellifera* at Selected Ontogenetic Stages and Their Immunogenic Potential during Summer

**DOI:** 10.3390/pathogens13020122

**Published:** 2024-01-28

**Authors:** Abdulkadir Yusif Maigoro, Jeong-Hyeon Lee, Hyunjee Kim, Olga Frunze, Hyung-Wook Kwon

**Affiliations:** 1 Convergence Research Center for Insect Vectors, Incheon National University, Incheon 22012, Republic of Koreabeamed79@hanmail.net (H.K.);; 2 Department of Life Sciences, College of Life Sciences and Bioengineering, Incheon National University, Incheon 22012, Republic of Korea; jhl02532@naver.com

**Keywords:** honeybee, *Apis mellifera*, age, gut microbiome, *Lactobacillus*, *Gilliamella*, lipopolysaccharide

## Abstract

Honeybees (*Apis mellifera*) are pollinating agents of economic importance. The role of the gut microbiome in honeybee health has become increasingly evident due to its relationship with immune function, growth, and development. Although their dynamics at various developmental stages have been documented, their dynamics during the era of colony collapse disorder and immunogenic potential, which are connected to the antagonistic immune response against pathogens, need to be elucidated. Using 16S rRNA gene Illumina sequencing, the results indicated changes in the gut microbiota with the developmental stage. The bacterial diversity of fifth stage larva was significantly different among the other age groups, in which *Fructobacillus*, *Escherichia-Shigella*, *Bombella,* and *Tyzzerella* were unique bacteria. In addition, the diversity of the worker bee microbiome was distinct from that of the younger microbiome. *Lactobacillus* and *Gilliamella* remained conserved throughout the developmental stages, while *Bifidobacterium* colonized only worker bees. Using an in silico approach, the production potential of lipopolysaccharide-endotoxin was predicted. Forager bees tend to have a higher abundance rate of Gram-negative bacteria. Our results revealed the evolutionary importance of some microbiome from the larval stage to the adult stage, providing insight into the potential dynamics of disease response and susceptibility. This finding provides a theoretical foundation for furthering the understanding of the function of the gut microbiota at various developmental stages related to probiotic development and immunogenic potential.

## 1. Introduction

*Apis mellifera* (*A. mellifera*), otherwise known as social insects, are among the most important pollinators worldwide [1]. The health of *A. mellifera* colonies has been a major concern considering colony losses in recent decades due to colony collapse disorder (CCD) during various seasons including summer [2,3,4]. Thousands of hives appeared empty, with a massive disappearance of bees [5]. Various factors contribute to this incidence, including heavy use of pesticides, climate change, pathogen infection, and excessive abuse of antibiotics [6,7]. These factors affect honeybee behavior and feeding [8]. However, the honeybee gut microbiota is closely related to the host and provide the host with several advantages, including improving food digestion, pathogen defense, regulating honeybee behavior and immunity, and promoting development [9,10,11].

In arthropods such as honeybees, variation in the gut microbial climax community is linked to health and fitness instability [12]. Adult honeybees can harbor as many as 10^9^ bacterial cells; these cells constitute eight prevalent phylotypes that constitute up to 95% of the overall bacterial population [13]. Among these bacteria, *Lactobacillus* is usually the most abundant and widespread in the gut [14]. Nutrient status, such as the change from relying on pollen to nectar, social life, such as from nursing to foraging, and developmental changes, such as from larvae to workers, also contribute to the microbiota stability [15,16]. Honeybee’s division of labor varies with their development time. Between 4 days post-emergence (dpe) and 12 dpe, worker bees serve as nurses and transfer royal jelly secretions to younger and older bees [17]. Between 9 days and 30 days, workers leave from hives and then focus on foraging for pollen, nectar, and water [18].

Additionally, all honeybee castes progress through different life stages, which can cause microbial infections to various extents [19]. This not only changes the microbiome composition but also affects the innate and humoral immune responses [19]. While the gut microbiota changed with the host environment, age, and diet, the pathogens that make up a small part of the microbiome communities contribute to their disease susceptibility [9,20,21,22]. Therefore, the pathogens might affect honeybee development [23]. The role of nonpathogenic microbes has gradually been appreciated, while animal health is greatly influenced by the microbial community within the gut [24,25,26].

In the past, Dong et al. revealed changes in the gut microbiome at different developmental stages throughout the life cycle of honeybees but restricted the changes to only worker bees [27]. Thus, their research provided a theoretical basis enabling way for further exploration of the gut microbiome during different developmental stages. Additionally, previous investigations into bacterial communities acting as symbionts in the Korean honeybee gut have identified important bacteria, such as *Bombella* and *Lactobacillus*, at several developmental stages that help maintain host nutrients and inhibit honeybee pathogens [28].

Unlike mammals, insects depend entirely on their innate immune system regarding pathogen defense [29]. Innate immune defense consists of cellular and humoral responses [30]. The humoral defense system reacts to macromolecules, such as soluble antimicrobial peptides (AMPs) and reactive oxygen species (ROS), as well as reactive nitrogen species (RNS) in the extracellular humor. Four families of AMPs are induced in the honeybee haemolymph upon immune challenge by Gram-positive and Gram-negative bacteria [31,32]. Waldan et al. observed the expression of the AMPs induced by the honeybee gut microbial symbiont for immune system stimulation and proposed that variation in immune responsiveness correlates with age as well as microbiota differences [33]. On the other hand, the cellular defense system is based on cells circulating in the insect haemolymph [34]. These immune cells (hemocytes) can be activated by lipopolysaccharides (LPS) [35]. In addition, they play a vital role in insects’ defense against pathogens [36]. They are known to have diverse activities, such as phagocytosis, encapsulation, and nodulation, and importantly, their function depends on the developmental stage of the insects [37]. LPS present in all Gram-negative bacteria as the main component of their membrane gives them immunostimulant efficacy [38]. Its concentration helps activate immune cells (hemocytes) in response to pathogens [35]. This process is governed by essential enzymes, among which LpxL and LpxM serve to produce the hexaacetylated form of Lipid A (the LPS domain), which is associated with different immunogenic agents [39].

However, with the current CCD era in Korea, the microbial community associated with honeybee developmental stages needs to be further elucidated. As pathogens are often associated with CCDs in combination with other factors [40]. The microbial variation at each developmental stage might explain their immune system activation potential, leading to either a strong immune system response or a weaker immune response from the host organism. Therefore, in this study, we categorized the microbial communities of honeybees at four different developmental stages and used an in silico approach to predict their health risk potential based on their LPS-endotoxin production potential. These findings could lead to the use of valuable and important genetic resources for improving the health condition of bees through the use of probiotics.

## 2. Material and Methods

### 2.1. Honeybee Sampling

We monitored *A. mellifera* at four developmental stages—namely fifth stage larva, newly emerged bees, nurse bees, and forager bees from three strong healthy colonies—from a beekeeping facility in the coastal area of Incheon National University apiary, South Korea, in the summer season, June 2023. A section of each hive where the fifth stage larva was present was carefully cut, the larvae were transported to the laboratory in a container with a regulated temperature of 33 °C, and three of the fifth stage larva were each placed in a 15 mL tube (The samples did not come into contact with the nurse bees). Frames with pupae were collected and placed under incubator conditions at 33 °C and 55% humidity. After hatched (24 h later), freshly emerged bees were collected. Nurse bees were collected and engaged in nursing activities near the larva within the selected colony. The forager bees were sampled as they entered the hive through the hive entrance carrying pollen on their legs. All the collected samples were placed into 15 mL plastic containers and properly transported to the laboratory, where they were stored at −80 °C. Three individuals were used per repetition for the four groups, and all experiments were conducted in triplicate. All the samples were collected from three different hives. Sampling for nurse bee and forager bee was performed on one day to avoid delay, and enable proper establishment of the microbiota under the same conditions.

### 2.2. Gut Dissection from the Whole Intestinal Tract of Honeybees

The honeybee surface was sterilized with 100% ethanol; thereafter, the intestinal tract was dissected (esophagus to rectum). The chest of the bee was held firmly with the left hand, while the chip with the venom sac was grabbed with the right forceps, and the gut was removed and placed into a 1.5 mL centrifuge tube. All the separated guts from the three individuals at the three developmental stages were stored in 75% alcohol in a low-temperature freezer at −80 °C for DNA extraction. The whole gut of the honeybees was subjected to genomic DNA extraction. In the case of the fifth stage larva, the whole larva- was used without separating the gut, and DNA was extracted by homogenizing the whole larva [41].

### 2.3. DNA Extraction

Under aseptic conditions, the total DNA of the gut microbiota was extracted using a PowerSoil Kit (47014; QIAGEN, Hilden, Germany) according to the manufacturer’s instructions. The final DNA concentration and purity were determined with a NanoDrop 2000 UV–vis spectrophotometer (Thermo Scientific, Branchburg, NJ, USA). The DNA quality was checked by 1% agarose gel electrophoresis.

### 2.4. Sample Preparation, 16s Sequencing, and Taxonomic Analysis

For all the samples, the National Instrumentation Center for Environmental Management (NICEM, www.nicem.snu.ac.kr, accessed on 20 October 2023), Republic of Korea, performed the commercial PCR amplification, sample processing, and 16S rRNA gene sequencing. The samples were amplified using KAPA HiFi HotStart ReadyMix (kk2601; Roche, Basel, Switzerland), and the first row forward and reverse primers for the V3–V4 region of the 16S rRNA gene were used for amplification (Table S1). The following PCR procedure was used: 3 min at 96 °C; 30 cycles of 30 s at 96 °C, 30 s at 55 °C, and 30 s at 72 °C; and 5 min at 72 °C. All the PCR analyses were subsequently performed on 1.2% agarose gels to determine the band size and intensity. Ampure XP beads (A63882; Beckman, CA, USA) were used to purify amplified DNA from each sample. According to the DNA content and molecular weight, samples were pooled in identical quantities and utilized to create Illumina DNA libraries. The libraries were subsequently sequenced via Illumina MiSeq runs to obtain 2 × 300 bp paired-end reads.

### 2.5. Analysis in QIIME2

The sequencing data were analyzed using the Quantitative Insights into Microbial Ecology (QIIME2) pipeline [42]. The raw reads were denoized and trimmed using the DADA2 pipeline [43]. All the data were individually denoized before being merged for further analysis. The QIIME2 diversity plugin was used to construct the alpha and beta diversity indices. The sampling depth was set at 3800. The bacterial diversity of honeybees from different developmental stages was compared using the Shannon index. Using QIIME2, the Shannon diversity index for honeybee samples was calculated using bacterial OTU count data. Taxonomic analysis was performed with the SILVA database v138.1. The Kruskal–Wallis H test was used to compare the results among developmental stages. To discover significant differences in their bacterial profiles, pairwise PERMANOVA was performed with the “beta-group-significance” tool in QIIME2 [44].

### 2.6. Bacterial Profiles

At the phylum and genus levels, read count and abundance data for the bacterial OTUs were evaluated. Low-abundance taxa with a value of less than one percent were categorized as “ETC” from the dataset. The term “Unknown” at the phylum level refers to unclassified reads. The relative abundance of each bacterial OTU was subsequently calculated across all the samples. The average and maximum abundances of bacterial phyla and genera in the dataset were used to identify abundant bacterial phyla and genera.

### 2.7. Venn Diagrams

To establish which microbiota were present in each honeybee developmental stage, genus-level abundance data for the honeybee samples were used to construct presence/absence data. A genus was considered to be present if it was found in at least one sample from that group.

### 2.8. Microbiome Profiling Based on Gram staining

Using an in silico approach, the gut microbiome from the four developmental stages were grouped based on Gram positivity and Gram negativity. The term “others” was used to refer to unclassified bacteria, as well as taxa that could not be classified at least up to the genus level. UniProt (https://www.uniprot.org/, accessed on 5 December 2023) [45] was used to identify the presence of each endotoxin synthetic enzyme (LpxL and LpxM) among the identified Gram-negative bacteria at the genus level according to the adopted method [46].

### 2.9. Statistical Analysis

The statistical analyses included the Wilcoxon rank-sum test, PERMANOVA (permutational multivariate analysis of variance), Kruskal–Wallis H test for calculating the Shannon index. Species accumulation curve analysis, which included 95% confidence intervals, was analyzed for species overlap or confidence interval (https://chao.shinyapps.io/iNEXTOnline/, 27 December 2023). Analysis was performed using R version 4.1.0 in RStudio Version 1.4.1106 and QIIME2 [47].

## 3. Results

### 3.1. Reads Profiling for Microbial Community 

Next-generation sequencing (NGS) returned an average of 67,000 quality trimmed reads with a maximum sequence depth of ~4000 bp. Out of the 11 samples, the fifth stage larva had two samples with a total of 14,884 nonchimeric reads. Three samples were collected from freshly emerged bees, for a total of 24,328 nonchimeric reads. There was a total of 21,257 nonchimeric reads from the three nurse bee samples. Finally, a total of 22,055 nonchimeric reads were obtained from the three forager bee samples (Table S2). To assess the adequacy of sequencing depth in the samples, a rarefaction curve was generated using the Shannon index as a measure of alpha diversity. Each sample was represented by a distinct color. The resultant rarefaction curve provided evidence that 11 sample reads have been shown to a satisfactory depth (Figure S1). 

### 3.2. Gut Microbiota Diversity and Richness between the Age Groups

**Figure 1:**
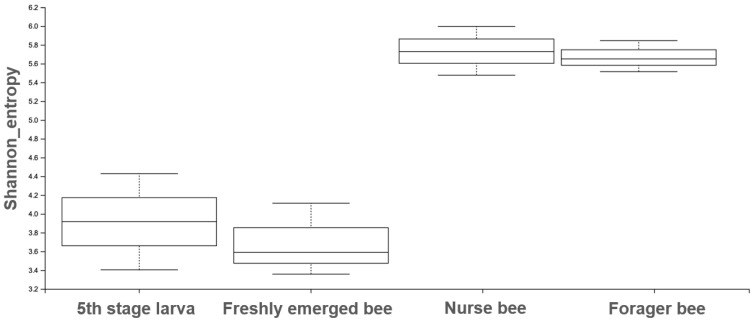
The Shannon entropy diversity index was calculated using statistical analysis to measure the degree of randomness of the microbiome diversity within a sample based on the species diversity and species richness of each sample. The box plot shows the diversity between the nurse bee/forager bee group and the fifth stage larva and freshly emerged bee groups.

**Figure 2:**
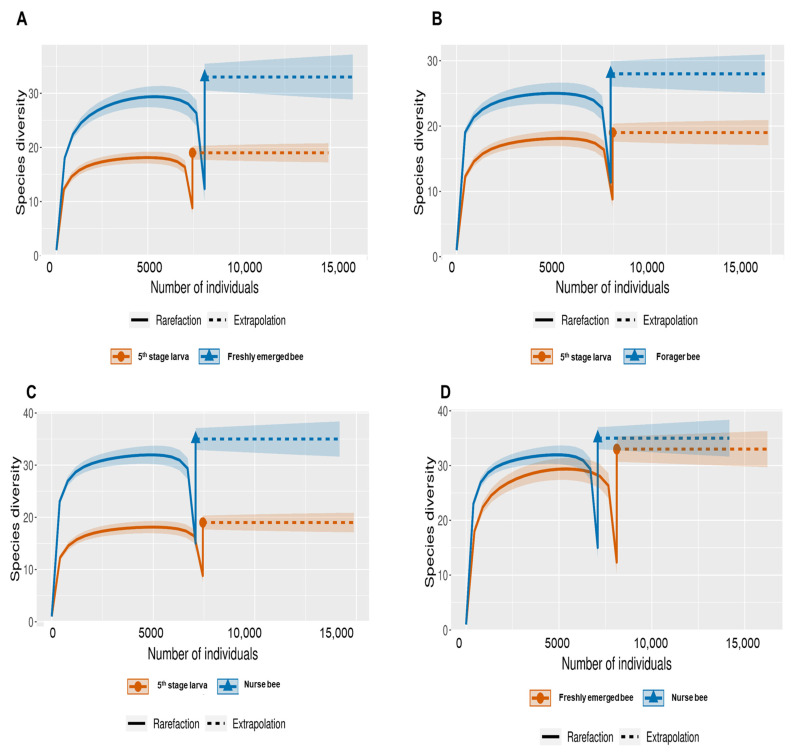

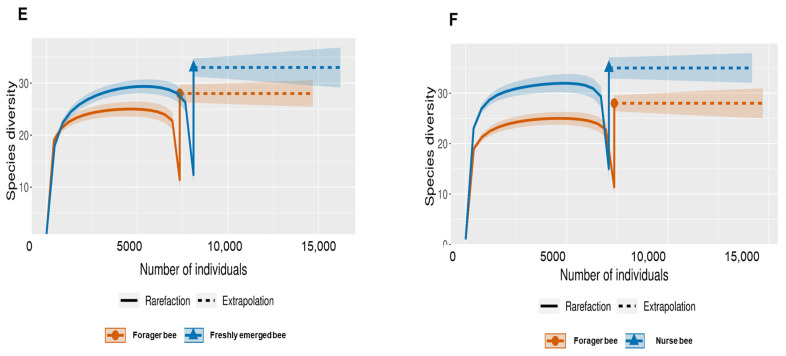
Species-size-based rarefaction (solid line) and extrapolation (dotted line) sampling curves with 95% confidence intervals (shaded areas) for different developmental stages. The developmental stages were compared with one another for specific overlap and/or significant difference identification. (**A**) Fifth stage larva and freshly emerged bee; (**B**) Fifth stage larva and forager bee; (**C**) Fifth stage larva and nurse bee; (**D**) Freshly emerged bee and nurse bee; (**E**) Forager bee and freshly emerged bee; (**F**) Forager bee and nurse bee.

**Figure 3:**
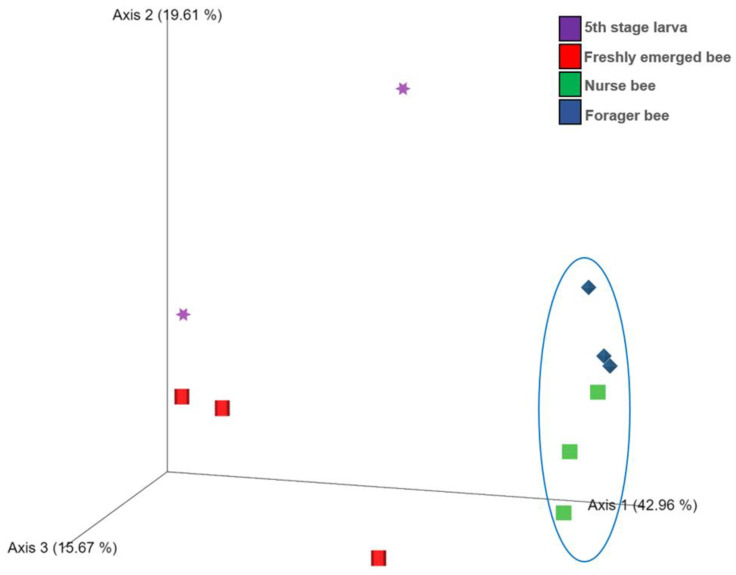
Score plot for principal coordinate analysis (PCoA) of the bacterial community compositions at the genus level in the *Apis mellifera* gut using a multivariate analysis method. The individual samples had different shapes and were color coordinated according to the developmental stage. Fifth stage larva, purple star; freshly emerged bee, red square; nurse bee, green rectangle; forager bee, blue diamond.

The Shannon index diversity shows that there was no variation between the fifth stage larva and freshly emerged bees but that a significant difference was observed between the younger honeybee (fifth stage larva/freshly emerged bee) and the worker honeybees (nurse bee/forager bee) groups (Figure 1). Furthermore, the Shannon index of a fifth stage larva from a forager bee was significantly greater than the Shannon index of a fifth stage larva from a freshly emerged bee. Similarly, the Shannon indices of freshly emerged bees from nurse bees and Forager bees were slightly significantly different. The Shannon index of the nurse bee did not significantly differ (Table 1). Further, species accumulation curves were used, including their 97% confidence intervals. This helped us determine species overlap and/or significant differences in relative abundance. The species diversity of the fifth stage larva was greater than that of the freshly emerged bees, forager bees, and nurse bees. Less species diversity was observed between the forager bees and nurse bees. On the other hand, species overlap was observed between the freshly emerged bees and the Forager bees, and stronger overlap was observed when comparing the Freshly emerged bee with the nurse bee. The species richness values that indicate the number of species were 20, 34, 36, and 29 for fifth stage larva, freshly emerged bees, nurse bees, and forager bees, respectively (Figure 2). The resulting clustering visualized by a principal coordinate analysis (PCoA) plot generated from weighted UniFrac distances showed variance in microbial communities depending on developmental stages (Figure 3). 

### 3.3. A. mellifera Gut Microbiota Abundance Distribution Based on Age

**Figure 4:**
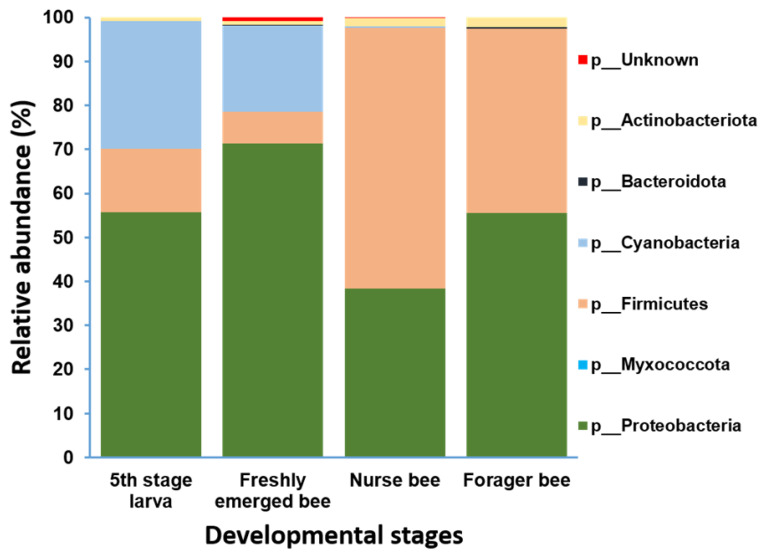
Microbiome composition (phylum level) of different samples in varying age groups. Phyla with a relative abundance > 1% were scaled up to 100% to visually represent the phylum-level diversity of all four groups. The microbiome relative abundances of fifth stage larva, freshly emerged bees, nurse bees, and forager bees are shown.

**Figure 5:**
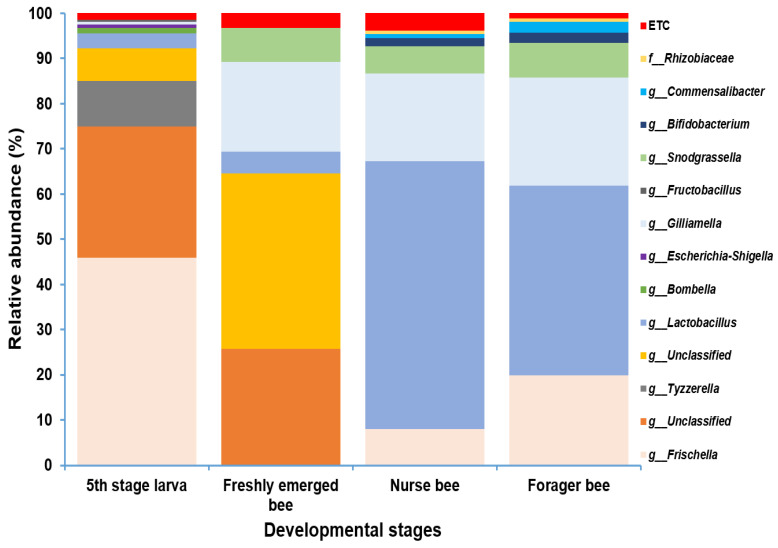
Microbiome composition (genus level) of different samples in varying age groups. Genera with a relative abundance > 1% were scaled up to 100% to visually represent the genus-level diversity of all four groups. The microbiome relative abundances of fifth stage larva, freshly emerged bees, nurse bees, and forager bees are shown.

**Figure 6:**
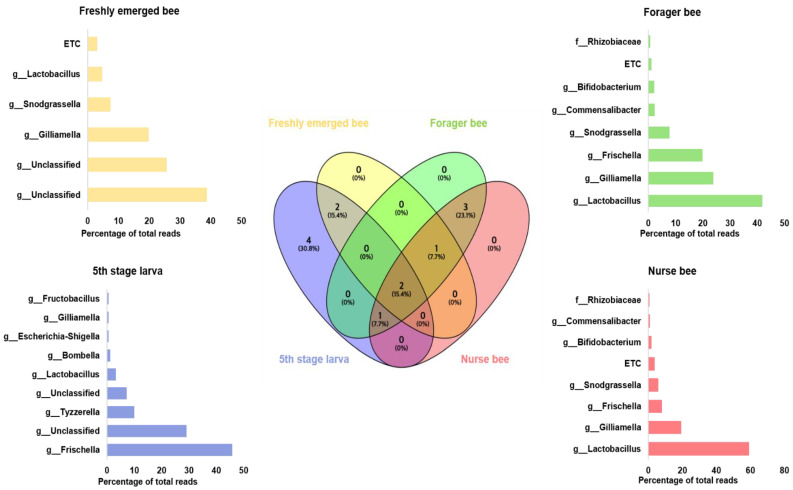
Venn diagram showing the microbiome distributions among the four groups at the genus level. The intercept values indicate shared genera, while the percentage (%) indicates the percentage of the genus among the total genus relative abundance. Linear discriminant analysis effect size (LEfSe) was performed. The bar size indicates the read size of each genus from each respective group. The microbiome percentage reads of fifth stage larva, freshly emerged bees, nurse bees, and forager bees are shown.

Gut microbiome analysis at the phylum level indicated that Proteobacteria and Firmicutes were widely distributed and predominant among all four groups. Cyanobacteria were highly abundant in the fifth stage larva and freshly emerged bee groups. Although Actinobacteria had a low relative abundance, they were equally widely distributed among the four groups (Figure 4). At the genus level, *Lactobacillus* and *Gilliamella* were ubiquitously present at the four developmental stages (Figure S2). However, *Lactobacillus*, *Gilliamella*, and *Frischella* were the predominant genera found among the nurse bees and forager bees (Figure 5). For fifth stage larva, *Frischella* and *Tyzzerella* were the predominant genera. In addition, *Gilliamella* dominated the gut of the newly emerged bees. A Venn diagram was constructed to visualize the microbiome distribution among the four groups (Figure 6). The fifth larval stage and freshly emerged bees shared no unique classified microorganisms. The nurse bees and forager bee shared three microorganisms: *Rhizobiaceae*, *Frischella*, and *Gilliamella*. In the fifth larval stage, the Nurse bees and Forager bees shared *Frischella* as a unique microorganism. 

### 3.4. Gut Microbiota of A. mellifera Based on Gram Positivity and Gram Negativity

**Figure 7:**
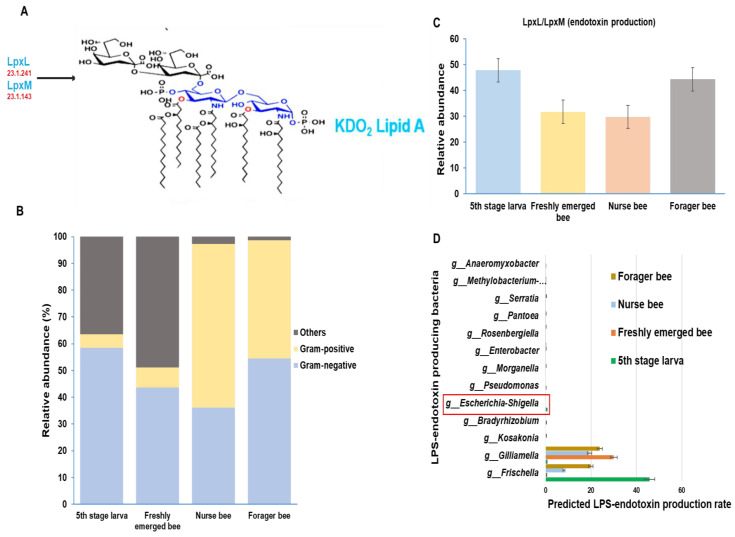
Schematic representation of lipopolysaccharide (LPS) biosynthesis, which results in the production of the inner part of LPS (Lipid A endotoxin) with the help of two essential final-stage synthetic enzymes (LpxL and LpxM). The enzyme commission numbers and products are shown in red and blue, respectively(**A**). Characterizing the *Apis mellifera* gut microbiome at four different developmental stages based on the presence of Gram-positive and Gram-negative bacteria (**B**). The predicted distribution of the LPS end products at the genus level and their relative abundances were calculated at each developmental stage (**C**). The list of LPS-endotoxin-producing bacteria and their production rates among the four developmental stages in *Apis mellifera.* The *E. coli* normally used as a model organism for LPS activity is highlighted in red (**D**).

The gut microbiome was analyzed and categorized based on morphological variation. There were more Gram-negative bacteria in the fifth larval stage and forager bee groups, with relative abundances of 58% and 54%, respectively. Among the nurse bees, Gram-positive bacteria were dominant, with a relative abundance of 61%. Among the Gram-negative bacteria, 43% were freshly emerged bees, while the majority were grouped as others. “Others” are groups of taxa that are either completely unclassified or unclassified up to the genus level and, as such, cannot be properly placed into either Gram-negative or Gram-positive groups (Figure 7B). Using the Gram-negative bacteria identified above, the presence or absence of essential enzymes for the final stage of LPS-endotoxin synthesis within the honeybee gut was analyzed. A greater endotoxin production potential was detected in the fifth larval stage and forager bee groups than in the other two groups (Figure 7C). Furthermore, a summarized distribution of each Gram-negative bacteria at the genus level was derived from the microbiota data (Figure 7D). The immunogenic potential of these peptides was predicted among *A. mellifera* developmental stages. The *E. coli* genus (*Escherichia-Shigella*), which is normally used as a model organism for LPS activity, was found only in the fifth stage larva group.

## 4. Discussion

This study provides evidence of gut microbiome alterations with honeybee developmental stage and further extends their biological role to predict their immunogenic potentials. In the past, the abundance and community structure of the microbiota change with the change in *A. mellifera* life cycle was proven, with the absence of core bacteria in larvae stage [48]. This might explain the high abundance of unclassified bacteria in the fifth stage larva and freshly emerged bees in addition to the lack of information in the database as well as unresolved sequencing problems. A significant correlation between the samples in nurse bee and forager bee groups was observed (Figure 3). Few samples from fifth stage larva and freshly emerged bee were clustered, while others were distanced. Overall, the samples from the younger groups were distanced from those in worker groups. These data indicated that honeybee development might significantly affect bee microbial community structure under natural conditions. The fifth stage larva exhibited a more varied microbiota within *A. mellifera* than in the other developmental stages [49]. According to our results, the fifth stage larva possessed four unique microorganisms not found in the three remaining age groups (Figure 6). The identified genera (*Tyzzerella*, *Fructobacillus*, *Escherichia-Shigella*, and *Bombella*) might be associated with larval age, lifestyle, immunity, and nutritional requirements. Honeybee endogenous *Fructobacilli* regulate larval and pupal storage proteins (hexamerin 70b), which contribute to developmental stages [50]. Acetic acid bacteria of the genus *Bombella* colonized various niches in beehives and are associated with larval protection against microbial pathogens [51]. It is worth noting that developing larvae have a discontinuous gut (the foregut is not connected to the hindgut) before pupation [52]. This makes them prone to many viral infections and is probably one of the reasons for having a different microbiome compared to that of the adult stage.

In honeybee workers, *Bifidobacterium* phylotypes were identified among the rarer members of the gut microbiota [48]. In our study, *Bifidobacterium* accounted for approximately 2% of the microbiome in both the nurse bees and the forager bees. Bacteria belonging to the *Bifidobacterium* genus are known as commensal microflora that inhibit the gastrointestinal tracts of humans and other animals [53]. These bacteria are nonspore-forming and nonpathogenic Gram-positive bacteria [54]. After their recent discovery in the stomach of *A. mellifera*, certain isolates of these bacteria exhibited antagonistic effects on Paenibacillus larvae, the causative agent of American foulbrood [55,56]. Therefore, members of the genus *Bifidobacterium* have been considered for use as probiotics for protecting honeybees from diseases [57].

On the other hand, *Gilliamella* species become increasingly abundant with age [58]. This finding is similar to the result obtained from this study, which showed a slight significant change in *Gilliamella* abundance, from a 19% abundance rate in nurse bees to a 23% abundance rate in foragers. It is worth noting that both task and age affect the gut microbiota by changing host physiology [58]. Furthermore, Copeland et al. identified the occurrence of ecological succession of the worker gut microbiota, with a clear species-level transition from nurse to forager bee [58]. This is similar to the result obtained in this analysis from species diversity with a 95% confidence interval. Greater diversity was observed between nurse bees and forager bees, while less diversity was observed between freshly emerged bees and nurse bees, with significant overlap highlighting the species transition from nurse to forager age groups. Regulation or dysregulation of the *A. mellifera* microbiome results in a reduction in *Lactobacillus* spp. and the expansion of *Gilliamella* spp. with age [58]. Although we did not observe this trend at the species level, a similar trend was recorded at the genus level, where the *Lactobacillus* abundance decreased from 59% to 41% for the nurse bees and forager bees, respectively.

Sometimes, from 12 to 21 days of age, honeybees spend more time on colony maintenance, such as comb building, later transition to nectar processing, and other tasks [59]. The nutritional requirements of these plant products vary among foragers (22–42 days) due to indirect access to pollen and flowers, hence affecting the microbiota [59]. However, the increase in the abundance of *Lactobacillus* in nurse bees might suggest a link between *Lactobacillus* and the production of larval food (Figure 5). Intriguingly, the presence of *Lactobacillus*, *Gilliamella*, and *Bifidobacterium* bacteria peaked during the adult stage, which corresponds to the stages of nectar and bee bread processing. This finding correlates with their functional capabilities, which are associated with carbohydrate breakdown. It was suggested that the lactic acid bacteria (LAB) group, consisting of *Lactobacillus* and *Bifidobacterium*, played a role in nectar processing and carbohydrate metabolism [60,61]. Divergent strains of *Gilliamella* exhibit different abilities to degrade pectin, an important plant pollen wall polysaccharide [60]. Even though these behavioral shifts might be due to changes in the microbiota composition, Martison et al. reported the characteristic phylotypes were maintained throughout the worker’s life [48].

*Lactobacillus* and *Gilliamella* were common genera with significant relative abundances among the four age groups. The conserved nature of these genera suggests their evolutionary importance for honeybee development. Earlier surveys have demonstrated that adult workers of the Asian honeybee harbor four major gut microbes, *Lactobacillus*, *Gilliamella*, *Snodgrassella*, and *Bifidobacterium*, suggesting that their abundance can be low at the larval stage and relatively increased at the adult stage [52,62]. This is consistent with our results (Figure 5). *Rhizobiaceae* (Rhizobium), the most well-known nitrogen-fixing bacterium, is recognized as an unusual bacterium found in the honeybee gut. Researchers believe that these are likely *Gilliamella*, which belongs to the order Rhizobium [63]. However, others have retained this genus as *Rhizobiaceae* [64]. However, unlike *Gilliamella*, *Rhizobiaceae* is found only in the Nurse and Forager bees.

Metagenomics analysis of a colony suffering from CCD revealed an increase in the relative abundance of *Lactobacillus* and a decrease in the abundance of *Bifidobacterium* [65]. This result revealed the negative effects of a reduced abundance of *Bifidobacterium* and a high abundance of *Lactobacillus*, which are thought to be protective in humans and other animals, including honeybees [55,66]. Pathogens are often associated with CCDs alongside various other contributing factors [40]. Therefore, microbial variation at each developmental stage might explain the immune system activation potential, leading to either a strong immune system response or a weaker immune response in the host organism. 

LPS present in all Gram-negative bacteria has immunostimulant efficacy [38]. Its concentration helps activate immune cells (hemocytes) in response to pathogens [35]. In this regard, both *Gilliamella* spp and *S. alvi* were reported as the two most abundant Gram-negative taxa in the honeybee gut [67]. They were antagonized by a pathogenic bacteria *S. marcescens* by which more *Gilliamella* were recovered in the process [67]. Extending that to *E. coli*, the effect of the pathogen was higher [67]. This highlights the strong effect of Gram-negative bacteria, especially *Gilliamella,* against prospective pathogens. *Gilliamella*’s higher abundance found in freshly emerged bees, followed by foragers, might project their varying immunogenic potential in the presence of pathogen. 

Powell et al. demonstrated that some Gram-positive members of the core microbiota can be acquired through contact with their hive surface, while Gram-negative members appear to be acquired through contact with nurse bees [68]. This indicates how the environment and social status can affect the gut microbiota composition. Given that the social role of *A. mellifera* varies with age, a greater abundance of Gram-negative bacteria was found among the forager bees (54%) than among the nurse bees (36%). 

The presence of some Gram-negative bacteria, such as *Gilliamella apicola*, was dependent on the presence of nurses, highlighting the correlation between the social role of honeybees and bacterial characterization [68]. The ability of a Gram-negative bacteria to trigger an organism’s immune response through lipopolysaccharide (LPS) endotoxin production can be utilized for early detection of infection because it induces an innate immune response [69]. Honeybee social interaction can be modulated by immune stimulation and as a result, LPS injection activates the immune system of the honeybee [70]. Our results show that microbial symbionts in the honeybee gut of foragers can influence the abundance of LPS. This suggests that the microbiota exerts a systematic immune effect, rather than being only localized to the gut.

Honeybees challenged with LPS lead to a reduction in longevity, which is related to age [71]. Furthermore, worker survival under immune challenge conditions is task dependent even though Nurse and Forager bees exhibit different gene expression patterns in response to LPS challenge [72]. This information can be of interest in fields that focus on how the immune system tackles challenges across developmental stages. Notwithstanding, excessive growth of these endotoxin-producing bacteria might be detrimental to honeybee health, which can be managed by having high contents of phenolic acids and internal hydrogen peroxide in the honeybee gut [73]. 

## 5. Conclusions

Developmental stages in *A. mellifera* not only contributed to changes in their microbiota but also influenced their immunogenic potentials. Our findings revealed that both LAC-producing bacteria (*Lactobacillus*) and pathogen–antagonist as well as LPS-producing bacteria (*Gilliamella*) remain conserved throughout the selected ontogenetic stages. Despite the limited number of samples, we suggest that the fifth stage larval microbiota distinctly varies because it does not harbor the core microbiome in the gut. Beekeepers should keep more effort on nurse bees as they display less immunogenic potentials among the workers, considering their important role for colony survival. While our analysis is in silico, we recommend experimental evaluation of LPS expression levels at varying developmental stages of different honeybee species in different seasons. Although the potential for LPS-stimulated pathogen-like immune response was observed in both *A. mellifera* and bumblebees, it was not evident at different developmental stages in either species [35]. Additionally, there is a need to identify the role of *Gilliamella* against opportunistic pathogens such as *S. marcescens* and viruses during varying developmental stages. This could provide more insights into both hive treatment and disease prevention through the modulation of gut microbiota to a healthy state. Finally, the conserved nature of *Lactobacillus* under natural conditions emphasizes its importance and reaffirms its potential status as a fecal microbiota transplantation agent for restoring normal microbial homeostasis.

## Figures and Tables

**Table 1:** The Shannon index was calculated as the *p-*value evaluated from the microbiome diversity within the respective samples. A *p-*value < 0.05 obtained using Kruskal–Wallis H test indicated statistical significance.

Group 1	Group 2	H	*p-*Value	q-Value
Fifth stage larva (n = 2)	Freshly emerged bee (n = 3)	0.33	0.56	0.67
Fifth stage larva (n = 2)	Forager bee (n = 3)	3.0	0.08	0.12
Fifth stage larva (n = 2)	Nurse bee (n = 3)	3.0	0.08	0.12
Freshly emerged bee (n = 3)	Forager bee (n = 3)	3.85	0.04	0.12
Freshly emerged bee (n = 3)	Nurse bee (n = 3)	3.85	0.04	0.12
Forager bee (n = 3)	Nurse bee (n = 3)	0.04	0.82	0.82

## Data Availability

The raw sequence and metadata generated were submitted to the NCBI BioProject database under accession number PRJNA1068260.

## Supplementary Materials

The following supporting information can be downloaded at: https://www.mdpi.com/article/10.3390/pathogens13020122/s1, Supplementary Table S1. Primer information for amplifying V3–V4 region. Supplementary Table S2. The table indicates the summary of the next-generation sequencing (NGS) analysis with a total of 11 samples. 5dayslarva samples (*n* = 2); freshly emerged bee samples (*n* = 3); forager bee samples (*n* = 3); nurse bee samples (*n* = 3). DADA2 output after removing the chimeric reads returned an average of 67,000 reads from the 11 samples. Supplementary Figure S1. Rarefaction curve plotted using alpha diversity metric, Shannon index against number of sequences per sample. Each colored line represents one biological sample. Supplementary Figure S2. Microbiome relative abundance distribution among each sample within the varying age groups.
