# Supplementary material for: Gut Microbiota of Apis mellifera at Selected Ontogenetic Stages and Their Immunogenic Potential during Summer
Source: Pathogens. 2024 Jan 28;13(2):122. doi: 10.3390/pathogens13020122 (PMC10893431; doi:10.3390/pathogens13020122)
Supplement: Supplementary file 1 [file pathogens-13-00122-s001.zip › Table S1.pdf]

Table S1. Primer for amplifying V3-V4 region

Primer	Sequence (5-------->3)
16S V3-V4_F	TCGTCGGCAGCGTCAGATGTGTATAAGAGACAGCCTACGGGNGGCWGCAG
	TCGTCGGCAGCGTCAGATGTGTATAAGAGACAGNCCTACGGGNGGCWGCAG
	TCGTCGGCAGCGTCAGATGTGTATAAGAGACAGNNCCTACGGGNGGCWGCAG
	TCGTCGGCAGCGTCAGATGTGTATAAGAGACAGNNNCCTACGGGNGGCWGCAG
16S V3-V4_R	GTCTCGTGGGCTCGGAGATGTGTATAAGAGACAGGACTACHVGGGTATCTAATCC
	GTCTCGTGGGCTCGGAGATGTGTATAAGAGACAGNGACTACHVGGGTATCTAATCC
	GTCTCGTGGGCTCGGAGATGTGTATAAGAGACAGNNGACTACHVGGGTATCTAATCC
	GTCTCGTGGGCTCGGAGATGTGTATAAGAGACAGNNNGACTACHVGGGTATCTAATCC
