# Supplementary material for: Gut Microbiota of Apis mellifera at Selected Ontogenetic Stages and Their Immunogenic Potential during Summer
Source: Pathogens. 2024 Jan 28;13(2):122. doi: 10.3390/pathogens13020122 (PMC10893431; doi:10.3390/pathogens13020122)
Supplement: Supplementary file 1 [file pathogens-13-00122-s001.zip › Table S2.pdf]

Sample-id	Input	Filtered	Percentage of input passed filter	Denoised	Merged	Percentage of input merged	Nonchimeric	Percentage of input nonchimeric
5th stage larva1	105397	81009	76.86	80923	69747	66.18	10478	9.94
5th stage larva2	86041	59373	69.01	59241	50206	58.35	4406	5.12
Freshly emerged bee1	90955	57467	63.18	57405	57120	62.8	10371	11.4
Freshly emerged bee2	84116	55308	65.75	55200	54790	65.14	10059	11.96
Freshly emerged bee3	77253	57566	74.52	57216	56169	72.71	3898	5.05
Nurse bee1	110258	82005	74.38	80826	76050	68.97	7063	6.41
Nurse bee2	117463	88331	75.2	86883	79889	68.01	7634	6.5
Nurse bee3	119125	88132	73.98	86597	79177	66.47	6560	5.51
Forager bee1	113475	86321	76.07	84946	78551	69.22	8208	7.23
Forager bee2	105013	78836	75.07	77438	71693	68.27	6890	6.56
Forager bee3	104392	80963	77.56	79507	72649	69.59	6957	6.66

Table S2. Raw read sequence per each sample. This include their input size, denoised sequenced and size of non-chimeric sequence.
