# Supplementary figures and images for: Gut Microbiota of Apis mellifera at Selected Ontogenetic Stages and Their Immunogenic Potential during Summer
Source: Pathogens. 2024 Jan 28;13(2):122. doi: 10.3390/pathogens13020122 (PMC10893431; doi:10.3390/pathogens13020122)

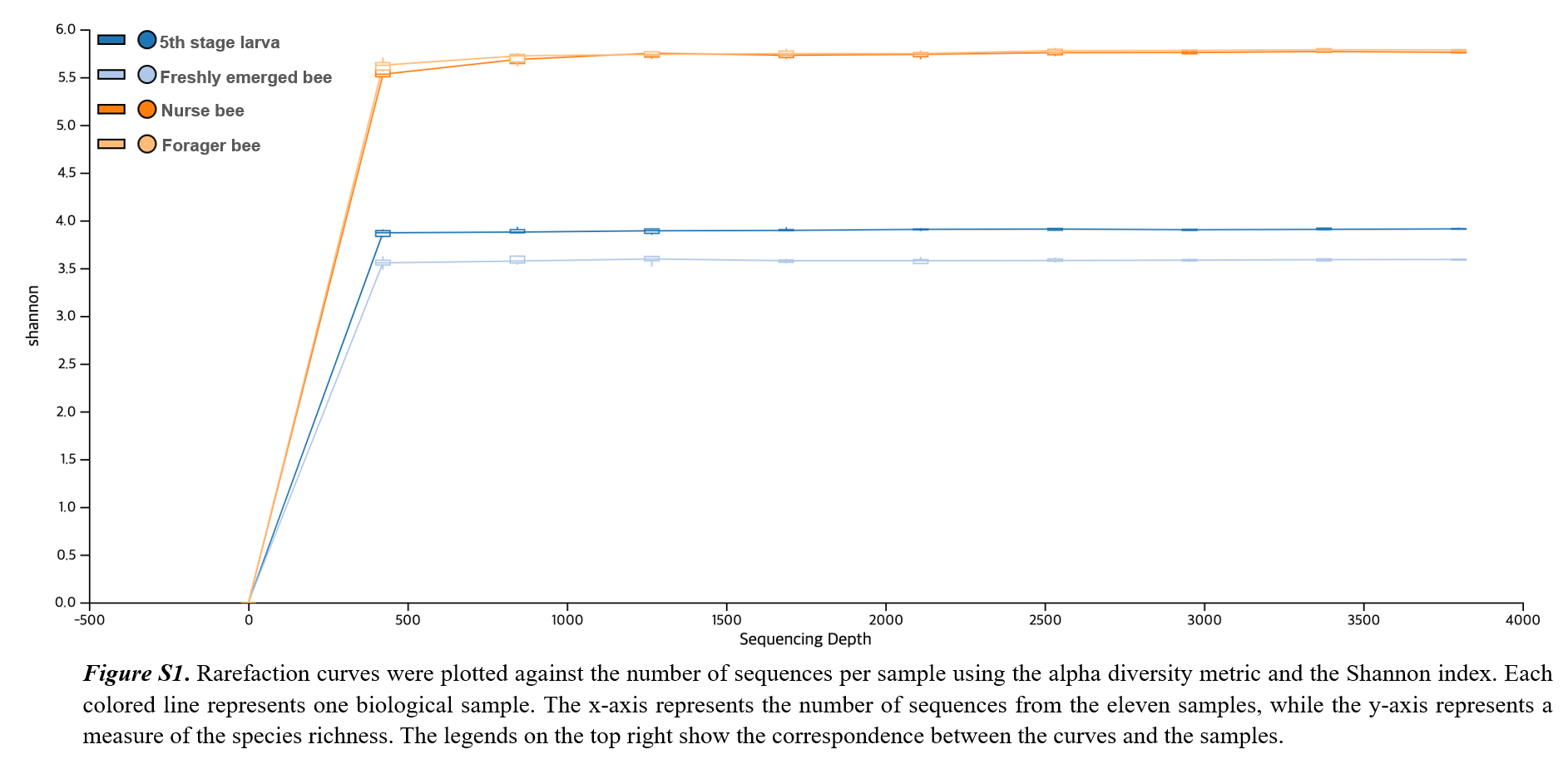

Supplement: Supplementary file 1 [file pathogens-13-00122-s001.zip › Figure S1.tif]

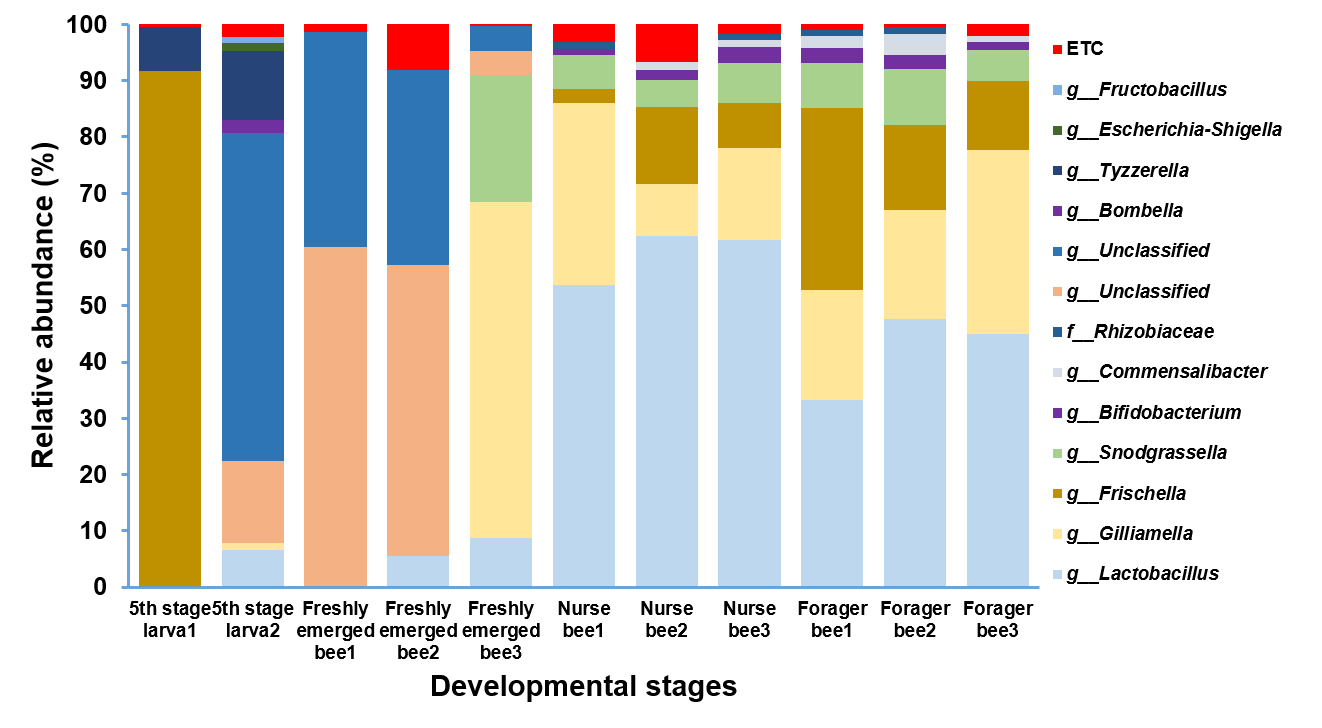

Supplement: Supplementary file 1 [file pathogens-13-00122-s001.zip › Figure S2.tif]
